# Photothermal sensitive nanocomposite hydrogel for infectious bone defects

**DOI:** 10.1038/s41413-024-00377-x

**Published:** 2025-02-14

**Authors:** Yanting Wu, Xi Xie, Guowen Luo, Jing Xie, Xiuwen Ye, Wanrong Gu, Anchun Mo, Zhiyong Qian, Chenchen Zhou, Jinfeng Liao

**Affiliations:** 1 State Key Laboratory of Oral Diseases & National Center for Stomatology & National Clinical Research Center for Oral Diseases, West China Hospital of Stomatology, Sichuan University, Chengdu, Sichuan China; 2 Department of Pediatric Dentistry, West China Hospital of Stomatology, Sichuan University, Chengdu, China; 3 Department of Biotherapy, Cancer Center and State Key Laboratory of Biotherapy, West China Hospital, Sichuan University, Chengdu, China

**Keywords:** Bone, Bone quality and biomechanics

## Abstract

Infectious bone defects represent a substantial challenge in clinical practice, necessitating the deployment of advanced therapeutic strategies. This study presents a treatment modality that merges a mild photothermal therapy hydrogel with a pulsed drug delivery mechanism. The system is predicated on a hydrogel matrix that is thermally responsive, characteristic of bone defect sites, facilitating controlled and site-specific drug release. The cornerstone of this system is the incorporation of mild photothermal nanoparticles, which are activated within the temperature range of 40–43 °C, thereby enhancing the precision and efficacy of drug delivery. Our findings demonstrate that the photothermal response significantly augments the localized delivery of therapeutic agents, mitigating systemic side effects and bolstering efficacy at the defect site. The synchronized pulsed release, cooperated with mild photothermal therapy, effectively addresses infection control, and promotes bone regeneration. This approach signifies a considerable advancement in the management of infectious bone defects, offering an effective and patient-centric alternative to traditional methods. Our research endeavors to extend its applicability to a wider spectrum of tissue regeneration scenarios, underscoring its transformative potential in the realm of regenerative medicine.

## Introduction

Massive bone structure defects, arising from severe trauma, complex pathologies, or extensive surgical resections, present a critical challenge to skeletal structural integrity and biological functionality, impacting a significant portion of the population, particularly high-risk groups such as the elderly and individuals with chronic diseases.^1^ The prevalence and severity of infectious bone defects, resulting from pathogen invasion through fractures or adjacent infections, exacerbate the situation. These infections trigger inflammation, lead to tissue destruction, and severely impair the bone healing process.^1–3^ The clinical treatments usually incorporate debridement and longtime use of antibiotics, which are heightened by the risk of severe complications and the growing concern of antibiotic resistance.^4–6^ As a result, the final treatment process faces extended treatment times and diminished outcomes.^7^ There is now a pressing need for pioneering, dual-functional treatment strategies that simultaneously address infection control and bone regeneration. Cutting-edge approaches, such as locally administered antimicrobials through polymer-based vectors and the integration of growth factors and antimicrobial agents into polymer scaffolds using tissue engineering techniques, are the focus of intensive research.^8,9^ Advancements in polymer materials and regenerative medicine hold the potential to transform the management of infectious bone defects, offering promising prospects for significantly enhanced treatment outcomes for patients.^10–12^

Researchers from around the world have made significant progress in enhancing bone regeneration while simultaneously killing bacteria.^13,14^ Initially, localized drug release mechanisms were introduced to reduce the side effects associated with the systemic use of antibiotic medications. However, the osteoinduction capacity of scaffolds cannot be improved with antibiotics alone. Additionally, the release of antibiotics from these systems is often uncontrolled, typically resulting in insufficient dosage.^15,16^ To address this, researchers have explored the controllable release of antibiotics, which allows for targeted bacterial eradication in situ.^17–19^ These materials are designed to respond to environmental stimuli, such as changes in pH, temperature, or the presence of bacterial enzymes, triggering the timely release of antibiotics. This responsiveness significantly enhances treatment efficacy by providing antibiotics precisely.

Inspired by the use of mild photothermal therapy (PTT) and the in situ stimulation of drug release, we have come out with our strategy for infectious bone repair. Our research introduces a therapeutic system that represents treatment progress in the treatment of bacteria killing and bone defects. This system integrates a thermal-sensitive hydrogel platform with mild photothermal materials, alongside a pulsed drug release strategy. The temperature-sensitive platform forms the foundation of this system, designed to respond to the mild PTT temperature at the site of the bone defect.^20,21^ This responsiveness enables the platform to provide controlled, localized release of therapeutic agents, ensuring pulsed drug delivery directly at the target site.^9^ The response release of drugs, enhances the antibacterial effectiveness, providing a potent mechanism to control infection without the risks associated with systemic antibiotic therapies. The key point of this innovative system is the pulsed drug released strategy under the mild PTT. By employing drugs at high concentrations for their bacteriostatic properties and at lower concentrations for promoting osteogenesis, the system addresses the challenges of infection control and bone regeneration.^22^ Meanwhile, the incorporation of mild photothermal treatment into this system helps to enhance the osteogenic induction of hydrogel with significant advancement in bone regeneration. This multifaceted approach not only promises enhanced efficacy, but also offers a more localized, efficient, and patient-friendly treatment alternative. Our approach integrates PTT with pulsed drug release mechanisms, a promising strategy in infectious bone defect treatment. The development of the temperature-sensitive platform highlights the advancements in material science within the realm of biomaterials.

The platform’s capacity to adapt to physiological temperature changes and control drug release marks a substantial advancement in smart biomaterials for medical applications. Its precise operation exemplifies the potential of engineering materials attuned to individual physiological needs, heralding new avenues in personalized medicine. This integration aims to maximize the therapeutic benefits, enhancing treatment effectiveness. The incorporation of mild PTT is innovative, offering a method to activate drugs directly at the defect site, thereby minimizing systemic side effects and improving patient outcomes.

**Fig. 1:**
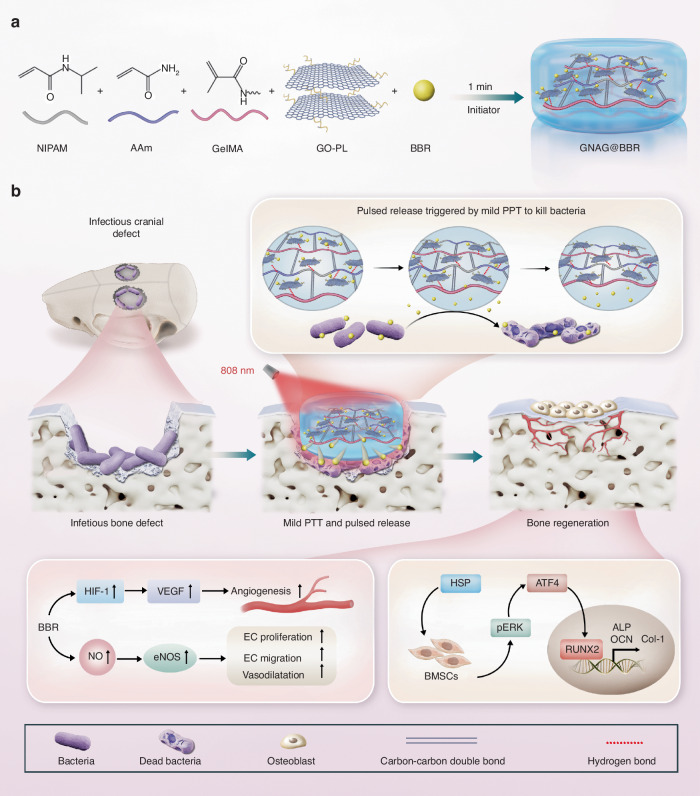
Scheme of the photothermal sensitive nanocomposite hydrogel to treat the infectious bone defect. **a** The preparation of the photothermal sensitive nanocomposite hydrogel. **b** The photothermal sensitive nanocomposite hydrogel for in vivo infectious bone defect repair in rats and its potential mechanism

In conclusion, this project represents a significant advancement in the field of regenerative medicine, particularly in the treatment of infectious bone defects. We have engineered a nanocomposite hydrogel system that effectively promotes both antibacterial activity and osteogenesis, marking a substantial progression in the field of biomedical engineering. The hydrogel system incorporates a temperature-sensitive polymer, mild PTT nanomaterial, and a pulsed drug release strategy to administer berberine (BBR), culminating in the preparation of GelMA/pNIPAM/pAAM/GO-PL@BBR (GNAG@BBR) nanocomposite hydrogels (Fig. 1a). This hydrogel was designed optimally to support infectious bone defect repair, initially utilizing mild PTT to trigger pulsed drug release for infection control and subsequently promoting bone regeneration (Fig. 1b). The in vitro and in vivo experiments demonstrated the photothermal sensitive nanocomposite hydrogel showed positive results for infectious control and bone repair. Therefore, our hydrogel system offers a promising new pathway to advance the treatment of infectious bone defects, setting the stage for future innovations in tissue engineering and regenerative therapy.

## Results

### Synthesis and characterization of GelMA/pNIPAM/pAAM (GNA) hydrogel

**Fig. 2:**
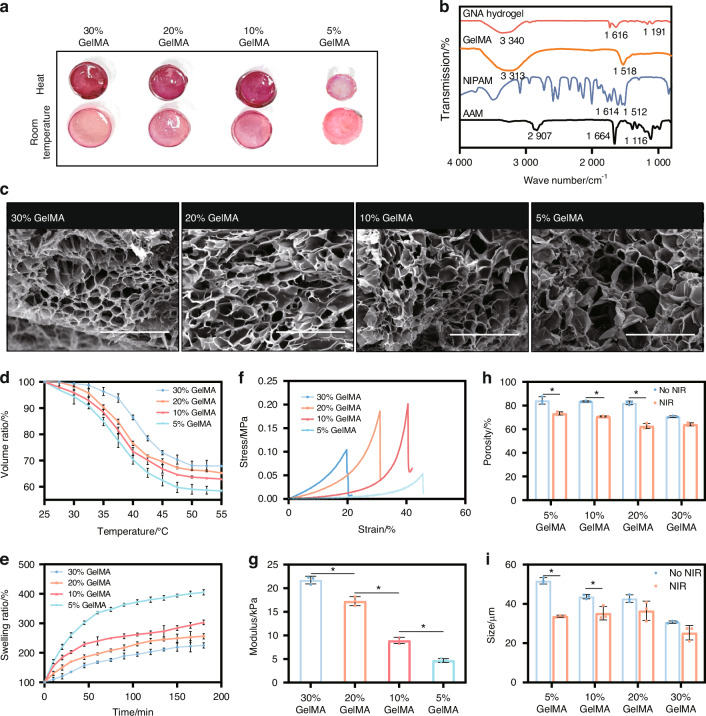
The selection of GelMA concentration in hydrogels. **a** General view of contraction behavior at elevated temperatures across different GelMA concentrations. **b** FT-IR analysis of hydrogel components. **c** SEM micrographs of hydrogel structures at various GelMA concentrations (scale bar = 500 μm). **d** Volume ratios at distinct GelMA concentrations. **e** Swelling ratios at distinct GelMA concentrations. **f** Stress-strain curves of hydrogels by GelMA concentration. **g** Young's modulus of hydrogels across concentrations. *P < 0.05. One-way ANOVA (*n* = 3). **h** Porosity and (**i**) pore sizes in hydrogels at different GelMA concentrations. *P < 0.05. Unpaired t-test, two-tailed (*n* = 3). Error bars represent mean ± SD

In our research, we synthesized a GelMA-based hydrogel using specific ratios of NIPAM to AAM for designed sensitive temperature, with a focus on modulating the concentration of GelMA. For morphological evaluation, samples were categorized into four groups based on GelMA concentration: 30%, 20%, 10% and 5% (Fig. 2a). FT-IR analysis was conducted to assess the chemical functionalities of the hydrogel components (Fig. 2b). The microstructure of the hydrogel was characterized using a Scanning Electron Microscope (SEM) (Fig. 2c). Key attributes, including volume ratio (Fig. 2d), swelling ratio (Fig. 2e), porosity, and pore size, were evaluated to determine the optimal GelMA concentration for our therapeutic applications.

We observed a significant enhancement in the mechanical properties of the hydrogel as the GelMA concentration increased, which was accompanied by a reduction in pore size and porosity. Specifically, Young’s modulus of the hydrogels increased to 21.7 kPa for 30% GelMA, 17.3 kPa for 20% GelMA, and was less than 10 kPa for both 5% and 10% GelMA (Fig. 2f, g).

However, such an increase in mechanical strength was offset by a decrease in pore size and porosity (Fig. 2h, i). At room temperature, pore sizes decreased with increasing GelMA concentration (from 51.76 µm for 5% GelMA to 30.74 µm for 30% GelMA). At 45 °C to ensure the shrink of GNA hydrogel, the reduction in pore size varied (34.8% for 5% GelMA, 14.5% for 20% GelMA, and 17.7% for 30% GelMA). Porosity also decreased with the increase of GelMA concentrations (from 84.35% for 5% GelMA to 70.64% for 30% GelMA).

For the photothermal-sensitive study, the hydrogel was heated to a mild photothermal temperature to observe the swelling change. Heating to 45 °C resulted in a reduction of porosity across all groups, with the most significant reduction observed in the 5% GelMA group (23.8%), suggesting that the intermediate ratio was more sensitive to heat. Both the volume ratio (VR) and swelling ratio (SR) decreased with increasing GelMA concentration, which is critical for applications requiring controlled swelling, such as drug delivery systems. The 20% GelMA hydrogel demonstrated an ideal volume variation, making it suitable for maintaining structural integrity in biomedical applications, such as bone defect repair. Consequently, we selected a 20% GelMA concentration as the optimal composition for the hydrogel.

### The characterization of GO-PL

**Fig. 3:**
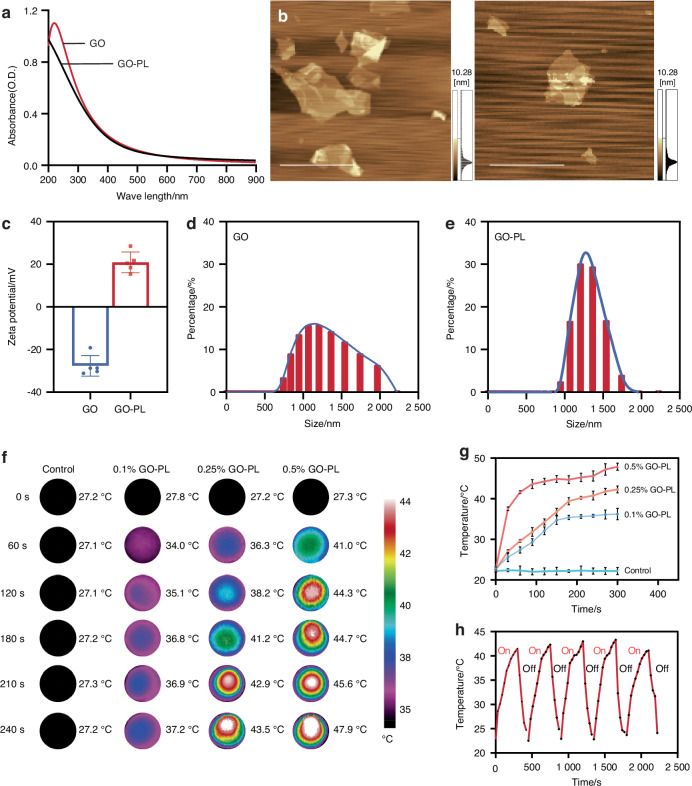
Analysis of GO-PL characteristics and influence on the properties of GNAG hydrogels. **a** UV spectroscopy of GO and GO-PL. **b** AFM of GO-PL and GO (scale bar = 5 μm). **c** *Zeta* potential (*n* = 3) and size distribution of GO-PL (**d**) and GO nanoparticles (**e**). **f** Thermal imaging of GNAG hydrogels with varied GO-PL concentrations. **g** Temperature variation curves for different GO-PL concentrations in GNAG hydrogels. **h** Five-cycle heating-cooling behavior of GNAG hydrogels at 0.25% concentration

We initially employed the EDC/NHS coupling method to graft PL onto GO. The successful synthesis of the GO-PL was confirmed through Ultraviolet (UV) spectrophotometry, which revealed the absence of the characteristic absorption peak for GO within the 200–300 nm range (Fig. 3a). This finding indicates the successful grafting of PL onto GO, leading to the formation of the GO-PL. Additionally, Atomic Force Microscopy (AFM) was utilized to examine the morphological characteristics of the GO-PL (Fig. 3b). This feature is expected to contribute to a more homogeneous dispersion within the nanocomposite hydrogel system. Particle sizes and *zeta* potentials of both GO and GO-PL were measured. GO exhibited a negative charge of −28.37 mV, while GO-PL demonstrated a positive charge of +20.9 mV (Fig. 3c). Particle size analysis revealed that GO-PL had a marginally smaller particle size than GO, though the difference was not significant. Furthermore, the introduction of PL led to a reduction in the PDI, from 0.492 to 0.197, indicating enhanced consistency (Fig. 3d, e).

### The optimal GO-PL concentration selection in hydrogel

A distinct correlation was observed between the concentration of GO-PL and the subsequent temperature increase during mild PTT in GelMA/pNIPAM/pAAM/GO-PL (GNAG) hydrogels. Higher GO-PL concentrations resulted in quicker temperature increases, with the 0.5% concentration reaching 48 °C after 5 min, which was too high for safe mild PTT (Fig. 3f, g). On the other hand, the 0.1% concentration remained below 40 °C, which is insufficient to trigger effective GNAG-mediated BBR release. The 0.25% concentration was found to be optimal, achieving a temperature range of 40–43 °C, which ensures both effective and safe therapeutic heating. Photothermal cycling tests demonstrated that the 0.25% GO-PL hydrogel maintained consistent efficiency over five cycles, indicating its reliability for repeated use (Fig. 3h).

### Characterization of nanocomposite hydrogel

**Fig. 4:**
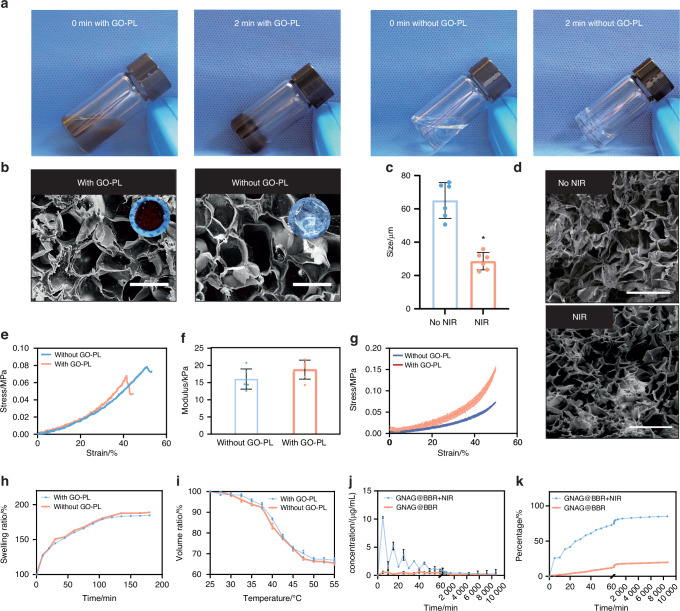
Comparison of hydrogels with and without GO-PL. **a** Overview of the appearances of both hydrogels. **b** SEM and the general view of the pore size change of the hydrogel before and after the PTT (scale bar = 100 μm). **c** Comparison of pore sizes of hydrogels before and after NIR exposure *P < 0.05. Unpaired t-test, two-tailed (*n* = 6). **d** SEM of hydrogels at room temperature or treated with mild PTT (scale bar = 500 μm). **e**, **f** Young's modulus for the hydrogel samples. **g** Stress-strain curves were observed over five compression cycles. Comparative analysis of the swelling ratios (**h**) and volume ratios (**i**), respectively. **j** Concentration curve depicting intermittent drug release from the hydrogel. **k** Cumulative release curve for the continuously released drug from the hydrogel

To assess the impact of GO-PL on the properties of nanocomposite hydrogels, a series of evaluations were conducted. No significant differences were observed following the incorporation of GO-PL (Fig. 4a). However, a noticeable change in color was evident upon the addition of GO-PL, suggesting a visual alteration in the hydrogel’s composition. The average pore size of hydrogel before GO-PL addition was 57.34 ± 15.49 μm, while the average pore size after GO-PL addition was 60.21 ± 23.59 μm. No significant differences in pore size were observed after the addition of GO-PL nanoparticles (Fig. 4b).

Notably, mild PTT induced significant changes in pore size, implying that the presence of GO-PL modulates the hydrogel’s thermal responsiveness (Fig. 4c). SEM observations confirmed that the microstructure of the hydrogel was preserved after NIR treatment (Fig. 4d). While the mechanical properties exhibited a moderate enhancement, Young’s modulus (*n* = 3) remained largely unchanged, indicating that GO-PL does not substantially affect the hydrogel’s mechanical integrity (Fig. 4e, f). Cyclic compression tests revealed a slight improvement in performance for the GNAG hydrogel in comparison to the hydrogel without GO-PL (Fig. 4g). Furthermore, the VR and SR remained unaffected by the incorporation of GO-PL (Fig. 4h, i).

### Drug release

After characterizing the nanocomposite hydrogel, the release properties of GNAG@BBR hydrogels were assessed over a period of 7 days. The early-phase drug release was monitored at 15 and 30-min intervals, while the latter phase was analyzed at 12-h intervals (Fig. 4j, k). Within the first 24 h, about 60% of the BBR was released. According to our study on the antimicrobial effect of BBR, it was found that the pulsed release of BBR was consistent with the minimum inhibitory concentration of BBR against *S. aureus* and *E. coli*.^10^ In other words, when applied in vivo, BBR maximizes its effect on bacteria in environments with inhibitory concentrations during the first 24 h.^18,19^ It helped to avoid the impact of infection on bone repair. After the initial release, lower concentrations of BBR helped in bone repair post-inflammation. Compared to a control group using a semi-permeable membrane, the GNAG@BBR hydrogel showed a pulsed BBR release, enhancing its potential for mending bone defects.

### Anti-bacterial assays

**Fig. 5:**
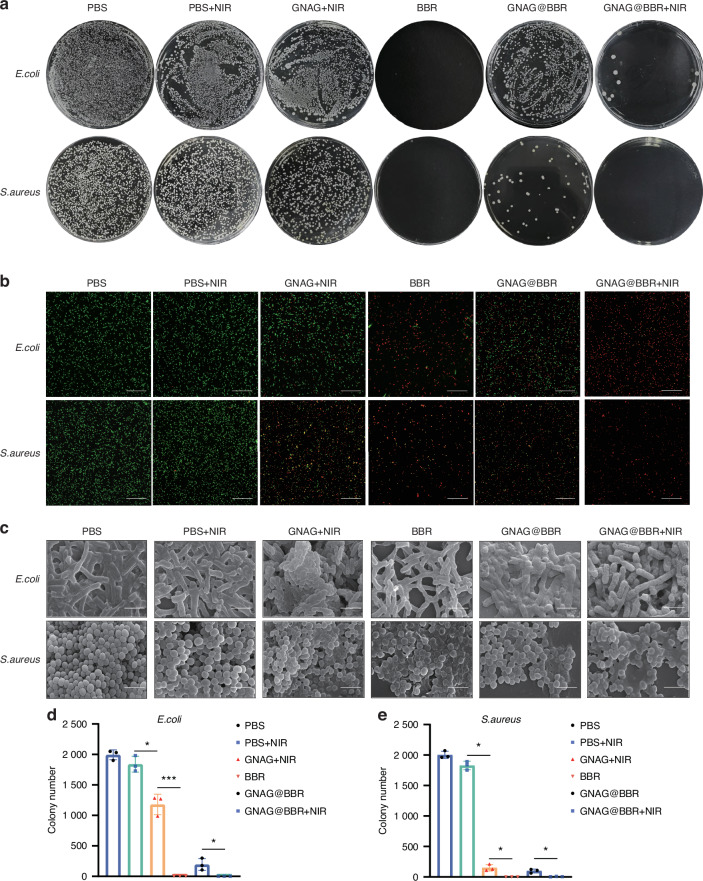
Anti-bacterial testing of GNAG hydrogel. **a** Assessment of in vitro bacterial inhibitory properties: the first row is *E. coli*, and the second row is *S. aureus*. The groups are from left to right: PBS, PBS+NIR, GNAG + NIR, BBR, GNAG@BBR, and GNAG@BBR + NIR. **b** Staining of live/dead bacteria, green color represents live bacteria, red color represents dead bacteria (scale bar = 40 μm). **c** SEM for observation of bacterial morphology (scale bar = 2 μm). **d**, **e** Colony counting of *S. aureus* and *E. coli* after different treatments. *P < 0.05; ***P < 0.001. One-way ANOVA (*n* = 3)

Our study evaluated the in vitro antimicrobial efficacy of dispersions and hydrogels fortified with BBR against *S. aureus* and *E. coli*. Both formulations effectively inhibited bacterial growth. A comparison between the PBS group and the PBS + NIR group showed that mild light and heat provided minimal antibacterial effects (Fig. 5a). The live/dead bacterial staining (Fig. 5b) also demonstrated bacterial fragmentation. With the fluorescent dyes NucGreen and EthD-III binding to DNA, fragmented cells lose their characteristic bacterial morphology, leading to DNA/RNA leakage and potential undetectability during the staining process.^20^ Furthermore, SEM was employed for the morphological examination of bacteria (Fig. 5c). In the BBR, GNAG@BBR, and GNAG@BBR + NIR groups, both *S. aureus* and *E. coli* bacteria displayed noticeable surface wrinkling. Moreover, a number of bacteria appeared fragmented or fractured, suggesting a compromise in cell wall integrity that leads to cell death. This accounts for the diminished bacterial count observed in both the GNAG@BBR + NIR and BBR groups. The hydrogel with BBR did not completely inhibit bacteria in the absence of NIR light, with a small population of *S. aureus* and *E. coli* to survive. However, the combination of hydrogel with BBR and NIR exposure (GNAG@BBR + NIR group) achieved a significantly enhanced antibacterial effect. This result underscored the synergistic effect of BBR and NIR light in bacterial eradication and suggested that mild PTT stimulation caused pulsed BBR release (Fig. 5d, e), thereby enhancing its antibacterial capability. The most significant effect was observed in the GNAG@BBR + NIR group, where the inhibition rate for *S. aureus* remarkably reached 100%. The live/dead bacterial staining results were in agreement with the quantitative antibacterial counting outcomes. Specifically, all *S. aureus* in the GNAG@BBR + NIR group were found to be non-viable, while only a few *E. coli* survived, highlighting the superior antibacterial efficacy of the GNAG@BBR + NIR group.

### Cell viability

**Fig. 6:**
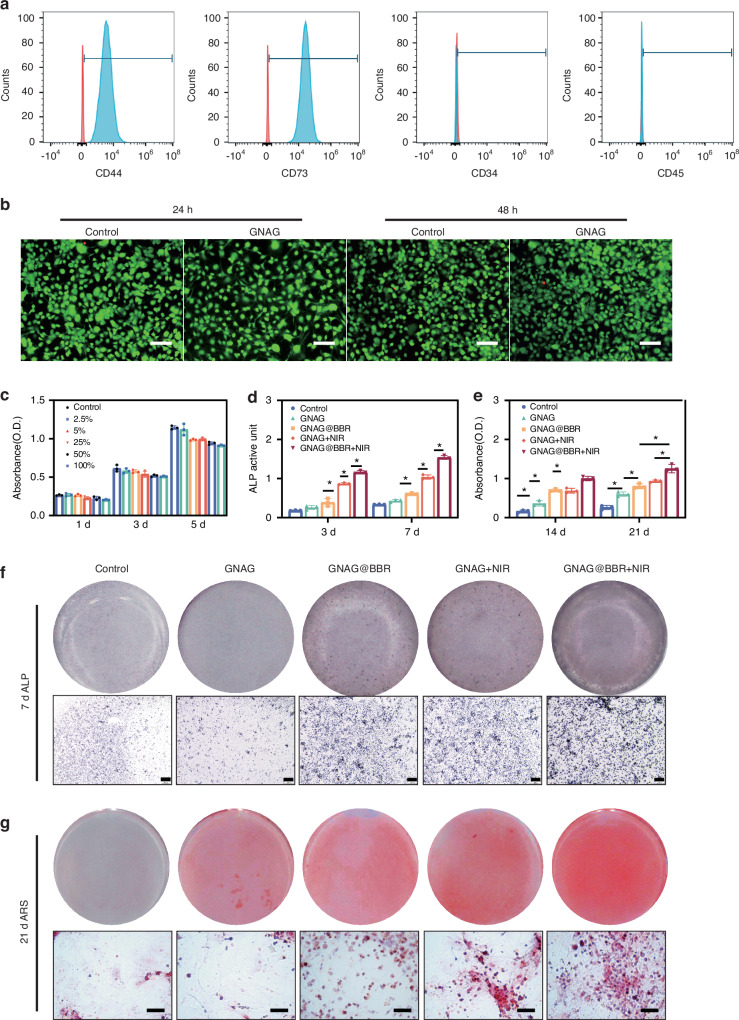
Evaluation of cytotoxicity and osteogenesis of bone marrow stem cells (BMSCs). **a** Identification of BMSCs: surface markers of BMSCs were detected by flow cytometry. Cells exhibiting CD44^+^, CD73^+^, CD34^-^, and CD45^-^ were identified as BMSCs. **b** Double staining of live/dead cells. The green regions represent live cells, while the red regions represent dead cells. (scale bar = 250 μm). **c** The survival rate of BMSCs was assessed on days 1, 3, and 5 using the CCK-8 assay. **d** Quantitative analysis of ALP activity was quantified on days 3 and 7. *P < 0.05. Two-way ANOVA (*n* = 3). **e** Quantitative analysis of Alizarin Red Staining (ARS) staining was performed on days 14 and 21. *P < 0.05. Two-way ANOVA (*n* = 3). **f** Representative images of ALP staining performed on day 7 (scale bar = 500 μm). **g** ARS staining on day 21 is presented (scale bar = 250 μm)

In the osteoblast differentiation study, BMSCs were extracted from 10-day-old Sprague-Dawley (SD) rats and purified to the third passage. The characterization revealed no expression of CD34 and CD45, but a positive expression of CD44 and CD73^15,16^ (Fig. 6a), indicating successful extraction of BMSCs. The cytotoxicity of the GNAGs was evaluated using the live/dead cell staining and Cell Counting Kit-8 (CCK-8). The live/dead cell staining at 24 h and 48 h post-inoculation confirmed that cell activity in the GNAG group remained consistent with the control group (Fig. 6b). Cytotoxicity assessments were performed at intervals of 1-, 3-, and 5-day post-inoculation using the CCK-8 test (Fig. 6c). On day 1 and 3, no significant differences in cell activity were observed. However, by day 5, a slight decrease in cell activity was noted with higher leachate concentrations, never exceeding 17% relative to the control group, within the acceptable threshold.^21^

### Osteogenesis assays of BMSCs with nanocomposite hydrogels

For osteogenesis assays, differentiation markers were evaluated as follows: Alkaline Phosphatase (ALP) at 3 and 7 days, ARS at 14 and 21 days. These markers were assessed across four groups: Control, GNAG@BBR, GNAG + NIR, and GNAG@BBR + NIR. Initial ALP assay results revealed no significant difference between the control and GNAG groups. However, notable differences (*P < 0.05, Fig. 6d, f) were observed among the other groups. The GNAG@BBR group promoted ALP expression on day 3 compared to the GNAG + NIR group, suggesting that higher BBR concentrations initially prioritized antibacterial activity. By day 7, with lower sustained-release BBR concentrations, its role in promoting osteogenesis became more evident.

To corroborate our findings on osteogenic differentiation, we employed both quantitative and qualitative ARS detection at 14 and 21 days. The quantitative assessment revealed variations in ARS expression between these time points, with all experimental groups exhibiting higher ARS levels on day 21 compared to day 14 (Fig. 6e, g). Significant statistical differences (*P < 0.05) were observed among all groups except GNAG@BBR and GNAG + NIR, confirming the bone repair efficacy of the nanocomposite hydrogel. The GNAG + NIR group showed more pronounced calcium nodule formation than the GNAG@BBR group by day 21, suggesting that mild PTT better promotes osteogenesis. Thus, light exposure was maintained for ~25 days (light irradiation every 5 days for 5 times).^1^ The findings spotlighted the exceptional potential of our nanocomposite hydrogel in promoting osteogenic differentiation, particularly in calcium nodule formation.

### Impact of nanocomposite hydrogel on biocompatibility and angiogenesis of HUVECs

**Fig. 7:**
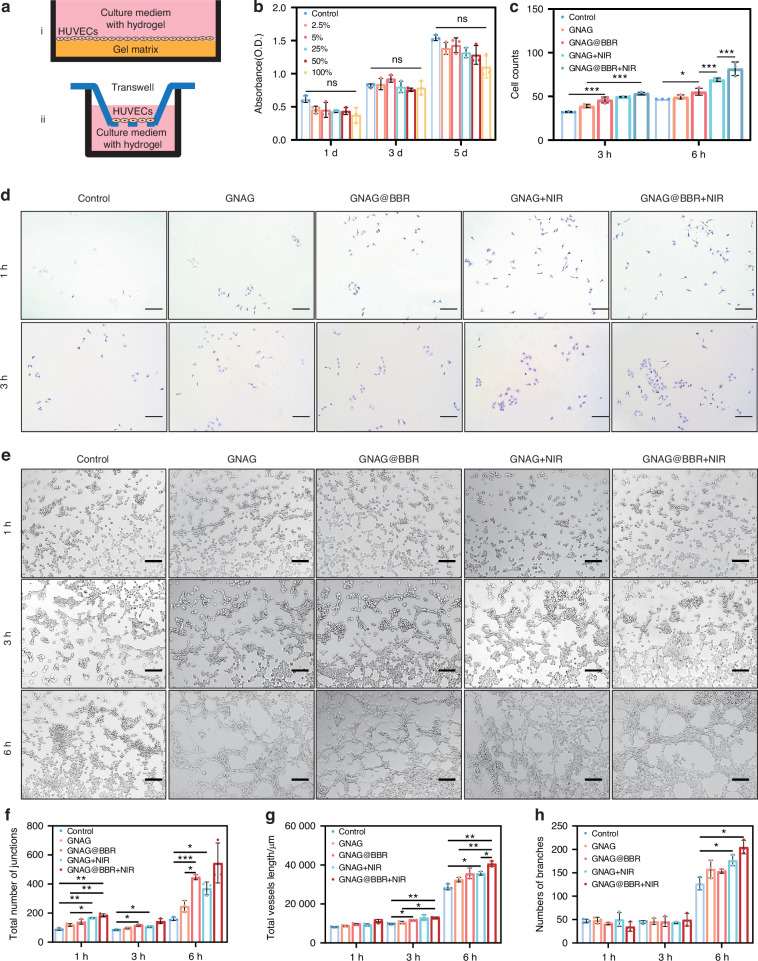
Biocompatibility and the angiogenesis of HUVECs. **a** The scheme of the procedure of angiogenesis (i) and migration (ii) of HUVECs. **b** The CCK-8 assay of HUVECs. **c** The cell counts of HUVECs for migration. **d** The crystal violet staining of HUVECs for migration (scale bar = 250 μm). **e** Tube formation in bright field (scale bar = 250 μm). The tube-formation analysis of HUVECs and the quantitative results of junctions (**f**), total length (**g**), and number of branches (**h**). *P < 0.05; **P < 0.01; ***P < 0.000 1. Two-way ANOVA (*n* = 3)

The biocompatibility of the nanocomposite hydrogels was evaluated using human umbilical vein endothelial cells (HUVECs). Figure 7a shows the experimental methods of the angiogenesis (i) and the migration of HUVECs (ii). Utilizing the CCK-8 assay, we observed no significant differences in cell viability between various concentrations of hydrogel leachate and the control group (Fig. 7b). This finding suggests that the nanocomposite hydrogel did not adversely affect HUVECs survival. Additionally, HUVECs migration was assessed using crystal violet staining under bright field conditions (Fig. 7c, d). Quantitative analysis of migrated cells revealed that the groups treated with GNAG + NIR and GNAG@BBR + NIR exhibited superior migration capabilities (Fig. 7c). Further experiments were conducted to measure the influence of the nanocomposite hydrogel on HUVECs’ capacity for angiogenesis (Fig. 7e). Notably, significant variations in angiogenic potential were observed across the treatment groups (Fig. 7f–h). Specifically, the nanocomposite hydrogel combined with mild PTT was found to be the most conducive to angiogenesis. These results imply that the nanocomposite hydrogel provides an environment that not only supports cell survival but also promotes the essential function of vascular formation.

### Mechanistic investigation of osteogenesis and angiogenesis

**Fig. 8:**
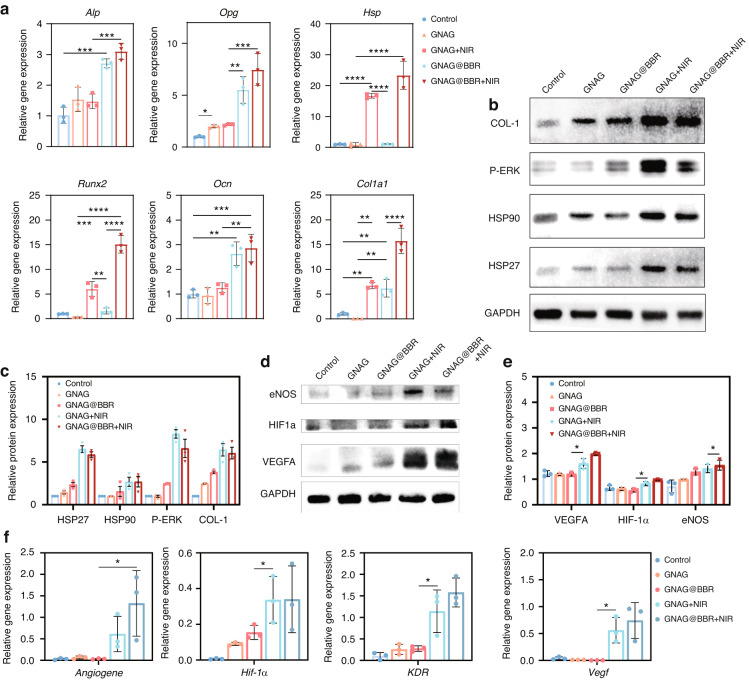
Angiogenesis and osteogenesis mechanism of GNAG hydrogel. **a** Relative gene expression profiles were obtained by qRT-PCR on day 7 following osteogenic differentiation. **P < 0.001; ***P < 0.000 1; ****P < 0.000 01. One-way ANOVA (*n* = 3). **b** Western blot analysis results on day 7 post-initiation of osteogenic differentiation in BMSCs. **c** Quantitative representation of western blot results. *P < 0.05. One-way ANOVA (*n* = 3). **d** Western blot analysis results at 24 h post-initiation of angiogenesis in HUVECs. **e** Quantitative representation of western blot results. *P < 0.05. One-way ANOVA (*n* = 3). **f** Relative gene expression profiles obtained by qRT-PCR at 24 h post-initiation of angiogenesis. *P < 0.05. One-way ANOVA (*n* = 3)

Our study revealed the significant roles of the GNAG hydrogel in enhancing osteogenesis and angiogenesis through key molecular mechanisms. Through qRT-PCR analysis, our research provided compelling insights into the role of BBR and mild PTT in osteogenesis (Fig. 8a). The genes associated with osteogenesis were significantly enhanced in groups exposed to BBR and photothermal treatments, which highlights the potential beneficial effects of these treatments in bone regeneration. Notably, *hsp* gene expressions saw an increase solely in the group subjected to photothermal irradiation, while other groups closely resembled the control group. This supports the hypothesis that mild PTT can modulate *hsp* gene expression, thereby influencing cellular osteogenesis. Further elucidation of the *runx2* gene, an effector of the *p-erk* pathway, bolstered this theory (Fig. 8b). Its upregulation in the group exposed to photothermal stimulation is consistent with existing research, affirming the hypothesis that mild PTT, through the orchestration of *hsp* and the p-ERK pathway, can indeed guide the osteogenic differentiation of BMSCs. Furthermore, the western blot procedure was used to measure the key proteins in the pathway (Fig. 8c).

To explore the molecular mechanisms underlying the GNAG hydrogel’s contribution to vascularization, we investigated several angiogenesis-related signaling pathways. Genes known to be pivotal in angiogenesis, including *angiogene*, *hif-1α*, *kdr*, and *vegfa*, were found to be upregulated in response to treatment (Fig. 8f). This upregulation was corroborated at the protein level (Fig. 8d). Quantitative analysis showed significant upregulation of these genes in the mild PTT groups (GNAG + NIR, GNAG@BBR + NIR), which was also evident at the protein level (Fig. 8e). These findings indicated that the GNAG hydrogel, especially when combined with PTT, enhanced vascularization by modulating the expression of key angiogenic genes and proteins. The enhanced pathways included the *angiogenesis* pathway, highlighted by the upregulation of the *angiogene*, which was critical for new blood vessel formation. The HIF pathway was also implicated, with increased expression of *hif-1α*, promoting angiogenesis and vascular remodeling under hypoxic conditions. The VEGF pathway was activated, as evidenced by the upregulation of *vegfa*, which stimulated new blood vessel growth and enhanced vascular permeability. Lastly, the VEGF-KDR pathway, with augmented expression of *kdr (vegfr-2)*, mediated signaling for endothelial cell proliferation and migration. These results indicate that the GNAG hydrogel, especially when combined with PTT, promotes vascularization by upregulating key angiogenesis genes and proteins, thereby supporting improved bone regeneration.

### GNAG degradation in vivo

**Fig. 9:**
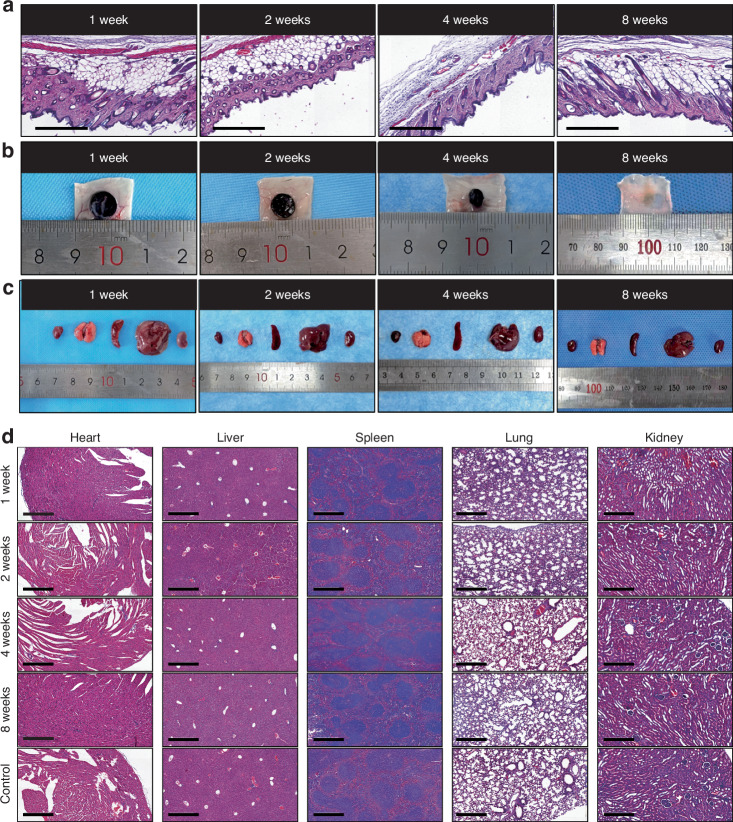
In vivo degradation and biocompatibility of nanocomposite hydrogels. **a** HE-stained subcutaneous tissues surrounding hydrogel samples were harvested from the mice’s back at 1-, 2-, 4- and 8-week intervals (scale bar = 250 μm). **b** Representative images of the implanted hydrogels at different time intervals. **c** General views of the major organs from the control and hydrogel group. **d** HE-stained images of major organs corresponding time intervals (scale bar = 250 μm)

To evaluate biocompatibility and degradation, GNAGs were subcutaneously implanted in mice and assessed at intervals of 1-, 2-, 4- and 8-week. The effects on tissues were investigated using HE-staining (Fig. 9a). Degradation was monitored through the observation of tissue disintegration (Fig. 9b). Major organs, including the heart, lung, spleen, liver, and kidney, were examined (Fig. 9c, d). No significant differences were observed when compared to controls, indicating excellent biocompatibility and potential clinical safety.

### In vivo infectious cranial defect repair in rats

**Fig. 10:**
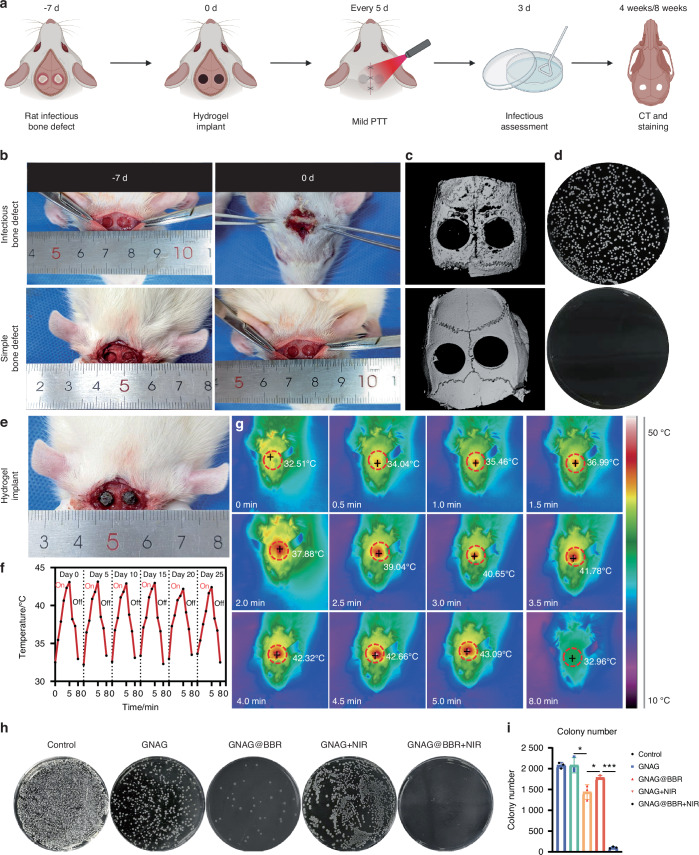
Overview and results of animal experiment procedures. **a** Scheme of the animal experiment processes. **b** Images depicting the infection status pre- and post-experiments. **c** Micro-CT scans of bone infection models, with (upper) and without (lower) bacterial presence. **d** Results of bacterial detection in soft tissues. **e** Image of the site post-hydrogel implantation. **f**, **g** Variations in temperature during mild PTT every 5 days, accompanied by infrared thermography images captured during the heating (0–5 min) and cooling (5–8 min) phases. **h**, **i** Counts and morphologies of bacterial colonies post a 3-day treatment period. *P < 0.05; ***P < 0.001. One-way ANOVA (*n* = 3)

A rat model was developed to study infectious skull defects and the timeline of animal experiments was shown in the scheme (Fig. 10a). The contrast between bacterially inoculated skull defects and non-infectious ones was observed both initially and after a period of 7 days (Fig. 10b). Micro-CT scans revealed an enhanced level of bone loss in the infected group, which extended beyond the intended model dimensions, resulting in the surrounding area resembling worm-eaten defects (Fig. 10c). An increase in bacterial colonies was observed in the infected groups compared to the non-infected groups (Fig. 10d). Following this, treatment with nanocomposite hydrogels was initiated, with the macroscopic evaluation depicted (Fig. 10e).

Upon model validation, rats were segregated into five groups for the in vivo repair of skull infection defects: Control, GNAG, GNAG + NIR, GNAG@BBR and GNAG@BBR + NIR. This experimental design facilitated the assessment of the effects of individual components. Mild PTT was administered every 5 days for a duration of 5 min to the designated groups. Infrared thermographic images were captured to monitor the procedure (Fig. 10f), and fluctuations in temperature were documented (Fig. 10g). The consistent photothermal performance highlights the suitability of the hydrogel. Three days post-implantation, skull samples and cranial soft tissue samples around the defect area were collected for the quantification of bacteria (Fig. 10h, i). A significant reduction in bacterial survival was observed in the BBR-containing groups. The GNAG@BBR + NIR group demonstrated almost no bacterial colonies three days post-surgery, attributable to the pulsed release of BBR. The GNAG and GNAG + NIR groups exhibited moderate bacteriostatic effects, with no statistically significant differences. The bacteriostatic effect in these groups is likely due to the pulsed release of the drug triggered by the initial stages of mild PTT.^17,18^

**Fig. 11:**
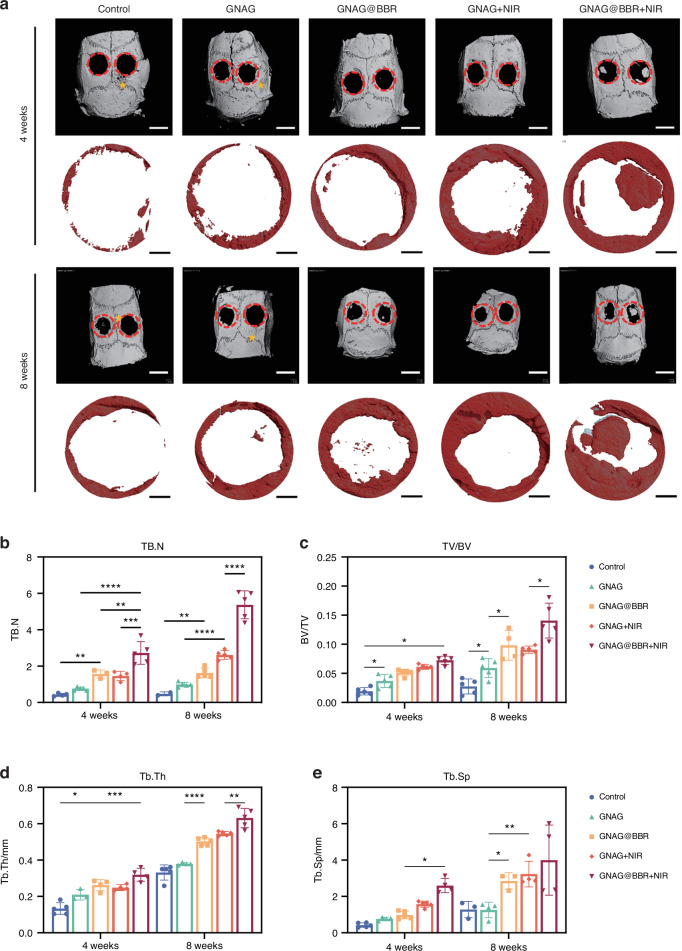
Micro-CT images and analysis of bone structure. **a** Reconstruction images at 4 and 8 weeks depict the defect areas (red circles) and additional bone defect regions 5 from bacterial erosion (asterisks) outside the primary defect area (scale bar = 100 μm). Quantitative analysis shows TB.N (**b**), BV/TV (**c**), Tb.Th (**d**) and Tb.Sp (**e**) at both time points. *P < 0.05; **P < 0.01; ***P < 0.001; ****P < 0.000 1. Two-way ANOVA (*n* = 5)

Micro-CT examinations of the skull were conducted at both 4-week and 8-week intervals (Fig. 11a). Through 3D reconstruction and detailed parameter evaluations, the control group exhibited minimal bone regrowth, while the GNAG + NIR group showcased superior bone regeneration. Furthermore, the GNAG@BBR and GANG@BBR + NIR groups displayed no destructive bone patterns, signifying the antimicrobial properties of the hydrogel and its role in preserving bone structure and managing infection. These observations emphasize the dual benefits of the nanocomposite hydrogel in controlling infection and preventing adjacent bone destruction. Notably, the GNAG@BBR + NIR group outperformed all other groups with a statistically significant difference (*P < 0.05), underscoring the efficacy of our fabricated GNAG system in accelerating the repair of infectious skull defects in rat models (Fig. 11b–e). In the quantitative analysis, the GNAG@BBR group demonstrated moderate effectiveness in controlling infection and promoting skull repair compared to the control and GNAG groups, but less than the NIR-treated groups. The BBR-infused GNAG exhibited stronger infection control, aiding bone repair and preventing the spread of infection and bone erosion in the defect area, in contrast to the GNAG + NIR group.

**Fig. 12:**
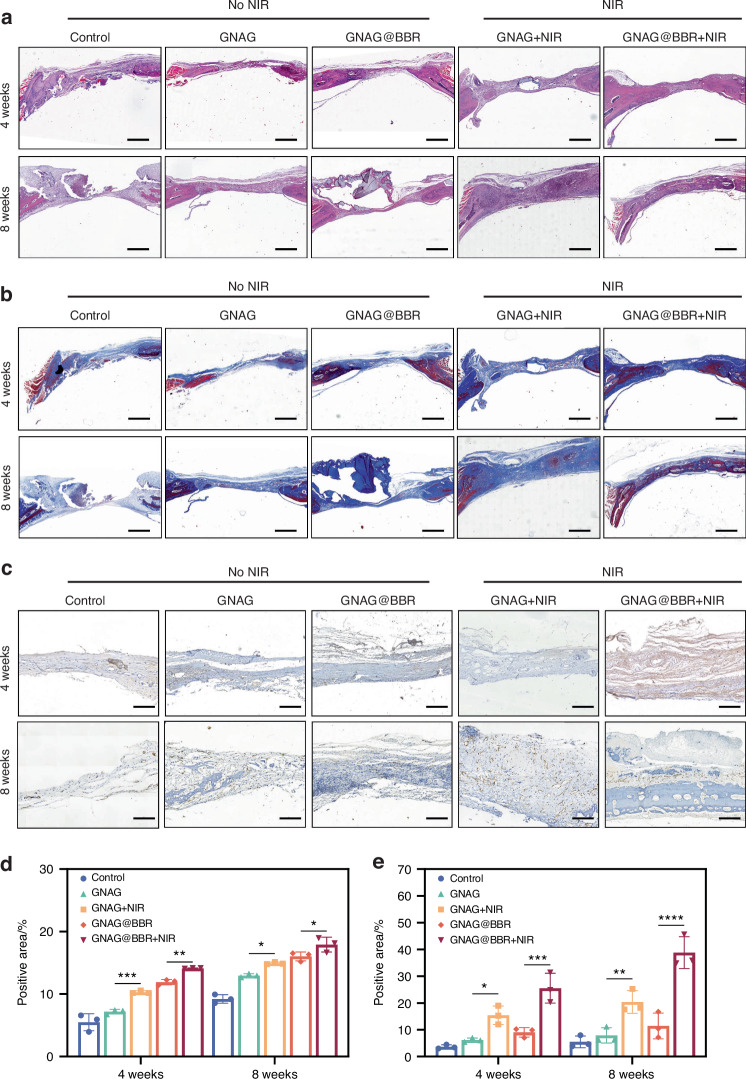
Histological analysis of bone repair at 4 weeks and 8 weeks post-implantation. **a** HE-stained sections at the respective time points. (scale bar = 100 μm). **b** Masson-stained sections, with an emphasis on collagen distribution (scale bar = 100 μm). **c** Immunohistochemical staining for CD31. The presence of CD31, indicative of endothelial cells within the tissue, is denoted by brown staining. Key annotations include HB (host bone), RB (repaired bone), H (GNAG hydrogel), and F (collagen fiber) (scale bar = 25 μm). **d** The quantitative positive areas of Masson-stained sections. **e** The quantitative of positive areas of CD31 immunohistochemical staining. *P < 0.05; **P < 0.01; ***P < 0.001; ****P < 0.000 1. Two-way ANOVA (*n* = 3)

HE and Masson staining were performed on the extracted skull sections to assess the extent and quality of bone repair (Fig. 12a, b). In the control group, the defect area was primarily composed of fibrous connective tissue, as indicated by Masson staining, with minimal bone regeneration observed. In contrast, sections from the PTT-treated group displayed significant new bone formation, as evidenced by both HE and Masson staining. The GNAG@BBR group exhibited partial bone repair, but less effectively than the PTT-treated groups. BBR enhances the bone healing capacity of the hydrogel, particularly when combined with mild PTT. The percentages of collagen formation were also evaluated (Fig. 12d).

Additionally, we investigated the vascularization capabilities of our treatments by performing immunohistochemical staining for CD31, a marker for vascular endothelial cells (Fig. 12c). The quantitative results are presented in Fig. 12e. The PTT groups demonstrated significantly higher CD31 expression compared to the control and GNAG@BBR groups, indicating an enhanced vasculogenic potential, which is vital for nutrient and oxygen exchange, thereby promoting bone regeneration. These findings suggest a potential correlation between increased vascularization and improved bone healing, meriting further exploration.

## Discussion

**Fig. 13:**
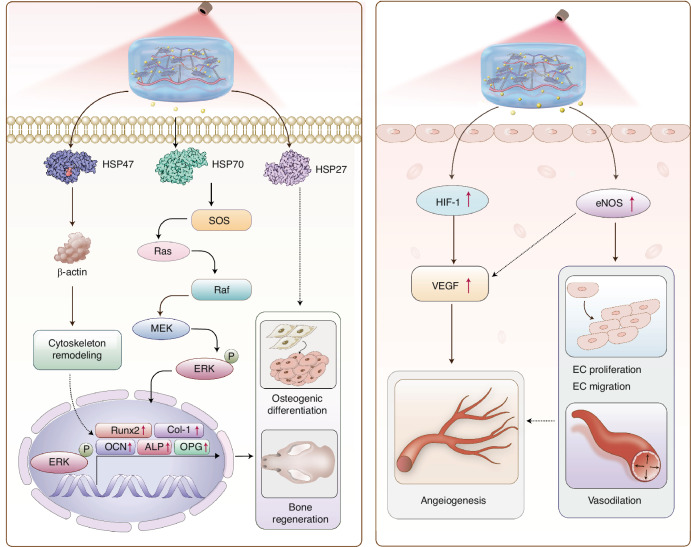
The potential mechanisms of photothermal stimulation of osteogenesis and angiogenesis

This study introduces a photothermal-sensitive nanocomposite hydrogel system, which has demonstrated significant efficacy in the dual management of infection and the facilitation of bone regeneration within a rat bone defect model. The research highlights the innovative convergence of photothermal effects with controlled drug release mechanisms, integrating temperature-responsive acceleration of pulsed drug release with the gentle application of PTT. The nanocomposite hydrogel exhibited remarkable photothermal responsiveness to near-infrared light, enhancing its osteoinductivity potential and substantially promoting bone regeneration. The proposed mechanism by which GNAG@BBR + NIR enhances bone regeneration and angiogenesis is delineated in Fig. 13.

Bone repair is influenced by three principal factors: osteogenesis, vascularization, and nerve repair.^22,23^ Angiogenesis has been established in the literature as both a prerequisite and facilitator of osteogenic repair, with infection control being equally essential for osteogenesis.^24^ Therefore, the simultaneous enhancement of angiogenesis and infection control is critical. To address this, a mild-PTT-triggered drug delivery system was developed.

In this study, BBR was selected for its infection-control properties and osteogenesis. Previous studies have indicated that the local application of antibacterial agents can prevent the overuse of antibiotics and the associated risk of liver and kidney damage.^3,7^ Moreover, certain plant extracts, which are clinically utilized, have demonstrated high biocompatibility and minimal toxicity to major organs.^25,26^ BBR was chosen for its established clinical use and its potential to promote bone regeneration across varying concentrations.^27^

Our recent findings suggest that moderate photothermal energy within the 40–43 °C range can significantly enhance skull defect repair in rats.^6^ This study uniquely combines gentle PTT with photothermal-induced pulsed drug delivery, underscoring the role of HSPs. HSP70, recognized for its role in promoting bone repair and osteogenic differentiation, was notably upregulated following photothermal stimulation. This upregulation facilitates osteogenic differentiation via the p-ERK signaling pathway. Additionally, our exploration of ER stress-induced signaling pathways sheds further light on the intricate molecular mechanisms underpinning osteogenesis, emphasizing the pivotal role of the endoplasmic reticulum in bone physiology. The inhibition of HSP70 protein expression has been shown to downregulate p-ERK expression and hinder bone formation, corroborating our experimental results.^28,29^ Further investigation into the roles of other members of the Heat Shock Protein family in this intricate process is warranted.

Our results are corroborated by numerous reports on the mechanism by which PTT promotes angiogenesis. We found that photothermal stimulation enhances the expression of angiogenesis-related genes in HUVECs. The successful upregulation of genes such as *hif-1a*, *kdr*, *vegf*, and others, promoted angiogenesis and HUVECs migration.^30^ This is particularly significant in the context of per osseous repair, as adequate angiogenesis during the early stages of osteogenesis provides a critical site for nutrient exchange and waste removal and is essential for bone mineralization in later stages. The local application of light and heat also enhanced blood circulation at the defect site, further supporting the reparative process.^31,32^

This study elucidates the molecular mechanisms that govern bone regeneration and angiogenesis. HSPs instigate the Ras/Raf/MEK/ERK pathway, leading to cytoskeletal remodeling and osteogenic differentiation. This process upregulates key transcription factors, including *runx2*, *col1a1*, *bglap(ocn)*, *alp*, and *opg*, which are essential for bone regeneration. Simultaneously, the elevated expression of *hif-1α* and *enos* enhances the levels of *vegfa*, which is crucial for endothelial cell proliferation, migration, vasodilation, and angiogenesis. These vascular events are critical for providing the regenerating bone with the necessary blood flow and nutrients.

In conclusion, our study has successfully engineered a photothermal sensitive nanocomposite hydrogel system that leverages mild PTT-triggered pulsed drug release for antibacterial activity, concurrently promoting bone formation. This innovative design facilitates the repair of infected bone defects by upregulating pivotal osteogenic and angiogenic factors, thereby controlling infection. The hydrogel has exhibited superior performance in the repair of rat skull defects. The integration of these methods presents a promising new solution for the treatment of bone defects, thereby heralding advancements in the fields of tissue engineering and regenerative therapies.

## Materials and methods

### Materials

N-(3-dimethylaminopropyl)-N’-ethylcarbodiimide, N-hydroxysuccinimide (NHS), carbodiimide (EDC), Poly-(L-Iysine) (PL), N-Isopropyl acrylamide (NIPAM), Graphene oxide (GO), acrylamide (AAM), and ammonium persulfate were procured from Aladdin, Shanghai. Yeast extract (OXOID, 4275289-02, UK), Sodium chloride (ChengShi, 20220912, CN), Tryptone (OXOID, 2927169, UK), Agar (BioFroxx, EZ67894140, EDU), phosphate Buffered Saline (Gibco, 812344, USA), Bacterial viability/toxicity test kit (UElandy, 220601L01-52, CN), Glutaraldehyde, 2.5% (Solarbio, 2308001, CN), 2, 2, 2-Tribromoethanol (MACKLIN, C14631680, CN) were also used. *Staphylococcus aureus* (*S. aureus*) and *Escherichia coli* (*E. coli*) were obtained from the State Key Laboratory of Oral Diseases, Sichuan University.

### Preparation of GNA hydrogel

GelMA, NIPAM, and AAM were each prepared as 20% stock solutions. GelMA concentrations in the hydrogel were adjusted to 30%, 20%, 10% and 5%. GO-PL was added at concentrations of 0.5%, 0.25% and 0.1%. APS and TEMED were added as the initiator and crosslinking agent at room temperature for 1 min, following precise guidelines for optimal reactivity and gel consistency. After polymerization, the gelatin was immersed in ddH_2_O to remove residual crosslinking agents and initiators. BBR was incorporated by soaking the gel in a BBR solution overnight.

### Synthesis and characterization of GO-PL

The synthesis of GO-PL commenced with the dispersion of 1 mL of graphene oxide (GO) in 20 mL of distilled water, referenced in literature.^16,17^ This primary mixture was subjected to sonication for a duration of 20 min to ensure a homogenous distribution of GO particles. Concurrently, solutions of 213 mg of 1-ethyl-3-(3-dimethylaminopropyl) EDC and 287 mg of NHS were each prepared in 5 mL of aqueous medium. In addition, 300 mg of PL was dissolved in 10 mL of water. Subsequently, the EDC and NHS solutions were combined with the GO dispersion. Following this, the PL solution was incrementally introduced, with the pH of the mixture being meticulously adjusted to a range of 4.5 to 5.5. To facilitate the complete integration of the components and to attain a consistent mixture, the resultant solution was sonicated for a period of 1.5 h while being continuously stirred at a rate of 600 r/min. Upon completion of the stirring phase, the GO-PL mixture was centrifuged and subjected to a series of three washes to remove any residual unreacted materials. The purified product was subsequently redispersed in double-distilled water (ddH_2_O) for subsequent applications.

For the purpose of characterization, AFM and UV absorbance spectroscopy were meticulously applied to the prepared samples. Initially, the samples were uniformly dispersed in water and then evenly spread onto mica sheets to ensure a consistent distribution. Once fully dried, the samples were mounted on the AFM stage for thorough microscopic examination. In tandem with AFM analysis, the UV absorbance properties of the samples were scrutinized using a Spectrophotometer U-3900H from Hitachi, Japan. By scanning across a spectrum ranging from 200 nm to 900 nm, comprehensive data regarding the absorbance characteristics of the samples within this wavelength range were obtained.

### Morphological assessment

The morphological attributes of the hydrogel were scrutinized after freeze-drying and sectioning the samples. Cross-sectional views of the hydrogel were examined using SEM (Apreo 2 s, Thermo Scientific, USA) to visualize the internal structure. The SEM not only provided images of the surface and interior but also allowed for the measurement of pore size and estimation of porosity. The pore dimensions were quantified using Image J software (National Institutes of Health, Maryland, USA), which facilitated precise pore size determination.

### Swelling ratio

The swelling behavior of hydrogel samples was assessed by comparing the change of the wet weight. The hydrogel samples were first lyophilized, and their initial weight was determined (W_0_). Subsequently, the samples were immersed in a physiological medium until they reached equilibrium, a process that typically spanned over 3 h. At regular time intervals, the samples were gently blotted with absorbent paper to remove surface moisture, after which the weight post-swelling (W_t_) was determined. The SR of the hydrogel was calculated using the following formula:

1 $${\rm{SR}}=({{\rm{W}}}_{t}-{{\rm{W}}}_{0})/{{\rm{W}}}_{0}$$

### Thermally induced volume variations in hydrogel

To determine the thermo-responsive behavior of the hydrogel, samples were fabricated under stringent conditions to ensure uniformity. The initial volume (V_0_) of the hydrogel was meticulously recorded. The hydrogels were then submerged in a graduated container filled with mineral oil, a medium that does not interfere with the hydrogel’s properties. A thermal cycle was applied by immersing the samples in a temperature-controlled water bath, with the temperature ranging from 25 °C to 55 °C. At each 5 °C increment, the samples were allowed to equilibrate before their new volume (V_t_) was measured using the displacement method. The VR was calculated using the formula:

2 $${\rm{VR}}={{\rm{V}}}_{t}/{{\rm{V}}}_{0}$$

This ratio illustrates the hydrogel’s sensitivity to temperature changes and its capacity to adapt, reflecting the volumetric response to thermal variations. This characteristic is pivotal for optimizing drug release efficiency through photothermal effects, changes in gel volume, and the dispersion of therapeutic agents. Specifically, under near-infrared irradiation, the GO-PL component warms up, reaches its Lower Critical Solution Temperature (LCST), triggers a phase transition, and induces gel contraction, thereby facilitating efficient drug delivery.

### Mechanical characterization

The mechanical integrity of the hydrogels was assessed using an Instron mechanical testing system (Norwood, MA, USA) at a crosshead speed of 1 mm/min under ambient conditions with a relative humidity of ~65%. Each sample was subjected to three mechanical tests to determine Young’s modulus and generate stress-strain curves. Furthermore, an anti-fatigue performance evaluation was conducted using a five-cycle compression test to measure the hydrogel’s resilience and durability under repetitive stress.

### In vitro drug release

The in vitro release profile of the drug from the hydrogel was evaluated following a systematic protocol. The hydrogel was immersed in 5 mL of distilled water to enable the gradual elution of the encapsulated drug. At specified time intervals, a 1 mL sample of the eluate was collected for drug concentration analysis, and an equal volume of fresh distilled water was added to the system to maintain sink conditions. The aliquots collected were then analyzed using an UV Spectrophotometer to quantify the amount of drug released over time.

### In vitro antibacterial testing

The in vitro antibacterial efficacy of the hydrogel was assessed by co-culturing bacteria with the hydrogel. The dilution of the co-cultured solution was prepared for colony counting. The experimental design involved six distinct groups: (1) PBS, (2) PBS + NIR, (3) GNAG + NIR, (4) BBR, (5) GNAG@BBR, and (6) GNAG@BBR + NIR. Both *S. aureus* and *E. coli*, standardized to a concentration of 1 × 10^8^ CFU/mL, were co-cultured with the materials from groups (1) to (6) in a 1:1 volume ratio. Notably, the specimens in groups (2), (3), and (6) were exposed to near-infrared light (808 nm near-infrared light laser, Leishi, MW-GX-808, 5 000 mW, Changchun, China) for 5 min at a power density of 2.2 W/cm^2^.

The co-culture conditions were strictly maintained at 37 °C in a 5% CO_2_ incubator for a duration of 4 h. Following the co-culture period, the solutions were diluted and spread, and the bacterial colonies were enumerated using a Chemiluminescence Apparatus (BIO-RAD, chemiDocTM MP, USA).

Additionally, the co-cultured solutions were analyzed using two complementary methods. Firstly, for the live/dead bacterial staining assay, the co-cultured solutions were centrifuged at 5 000 × *g* for 15 min to pellet the bacteria, and the supernatant was carefully aspirated. The bacterial cells were resuspended in a 0.85% NaCl solution and centrifuged again under the same conditions—a step that was repeated to ensure thorough washing (Centrifuge, Thermo Fisher, Pico 17, USA). The bacteria were then stained using a bacterial viability/toxicity kit and examined under a Confocal Laser Scanning Microscope (Olympus, Fluoview FV3000, JP) to assess cell viability and staining patterns. Secondly, for the bacterial SEM assessment, the co-cultured solutions were fixed with a 2.5% glutaraldehyde solution and stored at 4 °C overnight in a light-protected environment. The samples underwent a series of graded ethanol concentrations for dehydration, culminating in an SEM examination to visualize the bacterial morphology.

### Cell viability assessment

To initiate the cell viability assessment, BMSCs were cultured in a complete medium containing 10% fetal bovine serum (FBS) (Gibco, Australia) in αMEM (C12571500BT, Gibco, Suzhou, China). An extract representative of the hydrogel environment was prepared by soaking the hydrogel in αMEM medium. Given the potential concentration-dependent cellular response, multiple extract concentrations were prepared. The hydrogel was divided into six different groups based on the extract concentrations of 100%, 50%, 25%, 5% and 2.5%. A control group was also established where cells were exposed to the αMEM medium without any hydrogel extract. For the viability assessment, the osteoblastic cell line MC3T3-E1 cells were plated in 96-well plates and seeded with a density of 2 000 cells in each well. This standardized seeding density ensured uniformity across the experiment, enabling an accurate comparison between the different groups. The evaluation was conducted over a period of 7 days. At predetermined intervals, the cell viability was tested using the CCK-8 (C0037, Beyotime, Shanghai, China).

### In vivo biocompatibility and degradation

The animal experiments conducted in this study were ethically reviewed and approved by the West China College of Stomatology, Sichuan University (Ethical Review No. WCHSIRB-D-2021-132). We utilized 6-week-old postnatal BALB/c mice procured from Chengdu Dossy Experimental Animals Co., Ltd. (Sichuan, China). The prepared hydrogel, sterilized by overnight UV irradiation, was subcutaneously implanted into the backs of four mice under isoflurane-induced anesthesia to maintain statistical rigor. Following a 2-month observation period, with discrete intervals at the end of the first, second, fourth, and 8th weeks, the subjects were humanely euthanized. Subsequently, an extraction process was performed to precisely separate the dorsal subcutaneous tissue enclosing the implanted gel. The gross morphology was initially evaluated, serving as a crucial parameter for understanding the macroscopic interactions between the gel and the host tissue. The collected tissues were then preserved in a 10% solution of neutral formalin. The tissue was fixed and embedded in a supportive substance to facilitate subsequent procedures. The embedded samples were precisely sectioned, and the resulting histological analysis provided a microscopic view. This facilitated our evaluation of the impact of the new intervention on the cellular and tissue architecture.

### In vitro osteogenesis

In an investigation dedicated to in vitro bone formation, primary BMSCs were harvested from 10-day-old female SD rats, sourced from Chengdu Dossy Experimental Animals Co., Ltd. (Sichuan, China). Under anesthesia, the tibiae and femurs were carefully dissected, and the bone marrow was flushed with α-MEM to extract the stem cells. These cells were cultured until they reached passage 3. For the initiation of the experiment, cells were plated at a density of 1.0 × 10^5^ cells per well in 6-well plates, and the onset of treatment was determined when the cells reached ~80% confluence. To ensure experimental uniformity, the cells were categorized into five treatment groups: (A) Control, (B) GNAG, (C) GNAG@BBR, (D) GNAG + NIR, and (E) GNAG@BBR + NIR. Osteogenic induction was facilitated using a specialized medium. ALP activity was assessed at 3-day and 7-day intervals through staining and quantification. More advanced osteogenic markers were evaluated at 14-and 21-day post-induction using ARS, with quantification serving as the evaluative measure. Both ALP and ARS detection were performed according to the manufacturer’s instructions provided with the Beyotime kit (Shanghai, China). Furthermore, on the fifth day following treatment, cells were collected for additional analyses using quantitative real-time polymerase chain reaction (qRT-PCR) and western blot. Total RNA was extracted meticulously using the ES Science kit (ES-rn001, Suzhou, China), following the manufacturer’s protocol. This RNA was then reverse-transcribed into complementary DNA (cDNA) using the RNA-Quick Purification Kit (Yeason, 11141ES60, Shanghai, China). For qRT-PCR amplification, a series of specific primers were employed, targeting genes of interest including *sephrin (hsp)*, *bglap (ocn)*, *osteoprotegerin (opg)*, *alkaline phosphatase (alp)*, *collagen 1 a type 1 (col1a1)*, and *runt-related transcription factor 2 (runx2)*. The primers are shown in Table 1.

**Table 1:** Primer sequences for gene amplification in qRT-PCR analysis

Gene	Forward	Reverse
*Hsp70*	GCATTCTTACAATTGAAGAAGGTAT	ATCGGGGTAGCAAGAACAATCCATTT
*Ocn*	CCCTCTCTCTGCTCACTCTGCT	CTTACTGCCCTCCTGCTTGG
*Alp*	AGATGGATGAGGCCATCGGA	CCAAACGTGAAAACGTGGGA
*Runx2*	CATGGCCGGGAATGATGAG	TGTGAAGACCGTTATGGTCAAAGTG
*Col-1*	CTGGCAAGAACGGAGATGATG	ATCCAAACCACTGAAACCTCTG
*Opg*	CACGAGCCTTATCCCATTTGTAG	CTACCATAACCTACCCCTGCTTGT
*Kdr*	CCTCAATGTGTCTCTTTGCGCTA	TAAAGCCTATCTCGCTGTCCCA
*Vegfa*	ACATTGGCTCACTTCCAGAAACAC	TGGTTGGAACCGGCATCTTTA
*Hif-1α*	GGACGATGAACATCAAGTCAGCA	AGGAATGGGTTCACAAATCAGCA
*Enos*	GGACACAAGGCTGGAGGAG	ACACAGAAGGTTTCACAGGCA

### Impact of GNAG hydrogel on HUVECs biocompatibility and angiogenesis

HUVECs were provided by the State Key Laboratory of Oral Diseases. These cells were cultured in Dulbecco’s Modified Eagle Medium (DMEM) supplemented with 10% FBS and subsequently passaged. The substrate gel (Beyotime, Shanghai, China) was coated on 24-well plates by spinning until the gel was evenly distributed across each well. Cells were detached from the flask surface using trypsin and centrifuged at 1 000 r/min for 5 min at room temperature to pellet the cells. The cells were then resuspended in 2 mL of DMEM, and the concentration of HUVECs was determined by cell counting. The obtained HUVECs cell suspension was thoroughly mixed into the wells of the plate. Plates were incubated at 37 °C in a 5% CO_2_ atmosphere for 6 h. Images were captured using a microscope (Leica, Wetzlar, Germany).

### Western blot

Western blot was performed by standard techniques. The Omni-Easy™ One-step Color PAGE Gel Rapid Preparation Kit (Catalog number: PG212) was procured from Epizyme Biotech, Shanghai, China. The ECL chemiluminescent substrate, used for detection, was obtained from Abbkine Scientific, Georgia, USA (Product code: BMU102-CN). The ChemiDocTMMP Imaging System from BIO-RAD, California, USA, was employed for exposure. The following antibodies were used: GAPDH (Huabio Cat# ET1601-4, RRID:AB_3069615), HSP27 (Huabio Cat# ET1701-70, RRID:AB_3070235), HSP90 (Huabio Cat# ET1605-57, RRID:AB_3069720), P-ERK (Huabio Cat# ET1610-13, RRID:AB_3069896), Col-1 (Huabio Cat# ET1609-68, RRID:AB_3069877), VEGFA (Huabio Cat# ER30607), HIF-1α (Huabio Cat# HA721997), and Phospho-eNOS (Huabio Cat# HA721188).

### In vivo infected skull defect study

Female Sprague Dawley (SD) rats, aged 6 weeks, sourced from Dossy, Chengdu, were used as an in vivo model to establish infectious skull defects. Anesthesia was administered using 0.3% sodium pentobarbital, and the cranial region of the rats was surgically exposed. A concentrated suspension of *S. aureus* (0.5 mL containing 1.0 × 10^8^ cells/mL) was introduced into the cranial defect, after which the surgical incision was meticulously sutured. Each rat had ample access to food and water. A week post-surgery, rats were divided into five groups (*n* = 8 per group) after debridement: (a) Control; (b) GNAG; (c) GNAG + NIR; (d) GNAG@BBR; and (e) GNAG@BBR + NIR. The GNAG group received a nanocomposite hydrogel without BBR, while the GNAG + NIR group received the same hydrogel followed by mild photothermal treatment. The GNAG@BBR group received a BBR-infused hydrogel, and the GNAG@BBR + NIR group received both the BBR-infused hydrogel and PTT. Three days post-treatment, skull samples and cranial soft tissue samples near the skull defect areas were collected for bacteriological analysis. At 4- and 8-week post-operation, cranial tissues were evaluated using Micro-CT, Hematoxylin-Eosin (HE), Masson, and immunohistochemical staining.

### Statistical analysis

Statistical evaluation of the results was performed using a one-way analysis of variance (ANOVA) with a significance level set at *P* < 0.05. All data are presented as the mean ± standard deviation (SD). Tukey’s Honest Significant Difference test was conducted for post hoc multiple comparisons. GraphPad Prism (GraphPad, Boston, US) was utilized for all graph production.
